# Diisonicotinium penta­chloridoanti­monate(III) monohydrate

**DOI:** 10.1107/S1600536809019072

**Published:** 2009-05-23

**Authors:** Li-Zhuang Chen

**Affiliations:** a Ordered Matter Science Research Center, College of Chemistry and Chemical Engineering, Southeast University, Nanjing 210096, People’s Republic of China

## Abstract

In the title compound, (C_6_H_6_NO_2_)_2_[SbCl_5_]·H_2_O, the Sb^III^ atom exhibits a distorted square-pyramidal coordination geometry. The crystal structure is stabilized by inter­molecular N—H⋯Cl, N—H⋯O, O—H⋯Cl and O—H⋯O hydrogen bonds, forming an extended three-dimensional network.

## Related literature

For related structures, see: Bujak & Zaleski (1999 ▶); Feng *et al.* (2007 ▶); Shen-Tu *et al.* (2008 ▶).
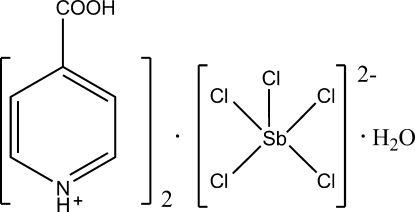


## Experimental

### 

#### Crystal data

(C_6_H_6_NO_2_)_2_[SbCl_5_]·H_2_O
*M* *_r_* = 565.25
Monoclinic, 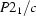

*a* = 10.334 (2) Å
*b* = 8.7319 (17) Å
*c* = 23.615 (7) Å
β = 106.98 (3)°
*V* = 2038.0 (9) Å^3^
*Z* = 4
Mo *K*α radiation
μ = 2.03 mm^−1^
*T* = 291 K
0.25 × 0.20 × 0.20 mm

#### Data collection

Rigaku SCXmini diffractometer
Absorption correction: multi-scan (*CrystalClear*; Rigaku, 2005 ▶) *T*_min_ = 0.61, *T*_max_ = 0.67
19258 measured reflections
4675 independent reflections
4071 reflections with *I* > 2σ(*I*)
*R*_int_ = 0.061

#### Refinement

*R*[*F* ^2^ > 2σ(*F* ^2^)] = 0.057
*wR*(*F* ^2^) = 0.085
*S* = 1.19
4675 reflections
227 parameters
H-atom parameters constrained
Δρ_max_ = 0.76 e Å^−3^
Δρ_min_ = −1.76 e Å^−3^

### 

Data collection: *CrystalClear* (Rigaku, 2005 ▶); cell refinement: *CrystalClear*; data reduction: *CrystalClear*; program(s) used to solve structure: *SHELXS97* (Sheldrick, 2008 ▶); program(s) used to refine structure: *SHELXL97* (Sheldrick, 2008 ▶); molecular graphics: *SHELXTL* (Sheldrick, 2008 ▶); software used to prepare material for publication: *SHELXL97*.

## Supplementary Material

Crystal structure: contains datablocks I, global. DOI: 10.1107/S1600536809019072/rz2323sup1.cif

Structure factors: contains datablocks I. DOI: 10.1107/S1600536809019072/rz2323Isup2.hkl

Additional supplementary materials:  crystallographic information; 3D view; checkCIF report

## Figures and Tables

**Table 1:** Hydrogen-bond geometry (Å, °)

*D*—H⋯*A*	*D*—H	H⋯*A*	*D*⋯*A*	*D*—H⋯*A*
O1—H1*C*⋯O1*W*^i^	0.85	1.67	2.520 (5)	175
N1—H1*B*⋯O4^ii^	0.86	2.45	3.031 (6)	126
O1*W*—H1*WA*⋯Cl3^iii^	0.85	2.71	3.378 (7)	136
O1*W*—H1*WB*⋯Cl5^iii^	0.85	2.54	3.241 (4)	140
O3—H3*A*⋯Cl5^ii^	0.85	2.19	3.034 (4)	175
N2—H2*A*⋯O2^iv^	0.86	2.41	2.988 (5)	125
N2—H2*A*⋯Cl2^v^	0.86	2.49	3.224 (4)	144
N1—H1*B*⋯Cl5	0.86	2.42	3.147 (4)	143

Symmetry codes: (i) 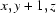; (ii) 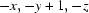; (iii) 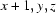; (iv) 
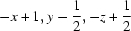
; (v) 
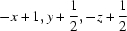
.

**Fig. 1.**
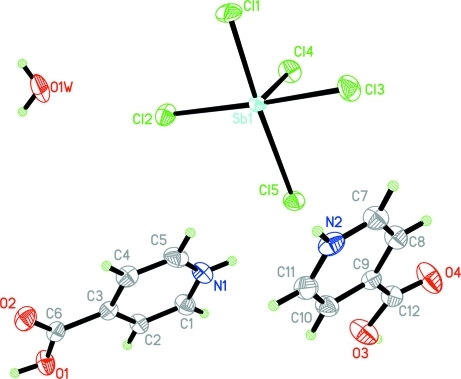
A view of the title compound with the atom-numbering scheme. Displacement ellipsoids were drawn at the 30% probability level.

**Fig. 2.**
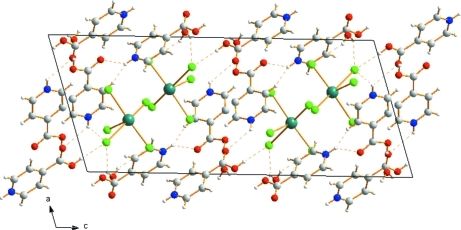
The crystal packing of the title compound viewed approximately along the b axis. Hydrogen bonds are drawn as dashed lines.

## supplementary crystallographic information

### Comment

Recently, the crystal structure of some halogenoantimonate salts has been reported (Feng *et al.*,2007; Bujak & Zaleski, 1999; Shen-Tu *et al.* 2008). As a contribution to this field, the synthesis and crystal structure of the title compound is reported herein.

The asymmetric unit of the title compound (Fig. 1) contains two protonated isonicotinic acid cations, a pentachloridoantimonate anion and a water molecule. The antimony(III) ion is in a distorted square-pyramidal coordination geometry, with the Sb—Cl distances ranging from 2.3642 (12) to 2.9002 (14) Å. This range of values is in agreement with that observed in N-methylpiperazinediium pentachloridoantimonate(III) monohydrate (2.4110 (10)–2.9112 (11) Å; Shen-Tu *et al.*, 2008) and slightly larger than that reported for bis(ethyldimethylammonium) pentachloroantimonate(III) (2.499 (4)–2.768 (4) Å; Bujak & Zaleski, 1999). The crystal structure is stabilized by intermolecular N—H···Cl, N—H···O, O—H···Cl and O—H···O hydrogen bonds (Table 1), forming an extended three-dimensional network (Fig. 2).

### Experimental

SbCl_3_, isonicotinic acid and 20% aqueous HCl in a molar ratio of 1:1:3 were mixed and dissolved in water by heating to 373 K forming a clear solution. The reaction mixture was cooled slowly to room temperature, crystals of the title compound were formed, collected and washed with dilute aqueous HCl.

### Refinement

All H atoms were fixed geometrically and treated as riding with C—H = 0.93 Å, O—H = 0.85 Å and N—H = 0.86 Å, and with *U*_iso_(H) = 1.2*U*_eq_(C, N) or 1.5*U*_eq_(O). The deepest residual electron density hole is located 1.47 Å from atom H5A.

### Crystal data

**Table:**

(C_6_H_6_NO_2_)_2_[SbCl_5_]·H_2_O	*F*(000) = 1104
*M**_r_* = 565.25	*D*_x_ = 1.842 Mg m^−^^3^
Monoclinic, *P*2_1_/*c*	Mo *K*α radiation, λ = 0.71073 Å
Hall symbol: -P 2ybc	Cell parameters from 4071 reflections
*a* = 10.334 (2) Å	θ = 3.1–27.5°
*b* = 8.7319 (17) Å	µ = 2.03 mm^−^^1^
*c* = 23.615 (7) Å	*T* = 291 K
β = 106.98 (3)°	Block, colourless
*V* = 2038.0 (9) Å^3^	0.25 × 0.20 × 0.20 mm
*Z* = 4	

### Data collection

**Table:**

Rigaku SCXmini diffractometer	4675 independent reflections
Radiation source: fine-focus sealed tube	4071 reflections with *I* > 2σ(*I*)
graphite	*R*_int_ = 0.061
Detector resolution: 13.6612 pixels mm^-1^	θ_max_ = 27.5°, θ_min_ = 3.1°
ω scans	*h* = −13→12
Absorption correction: multi-scan (*CrystalClear*; Rigaku, 2005)	*k* = −11→11
*T*_min_ = 0.61, *T*_max_ = 0.67	*l* = −30→30
19258 measured reflections	

### Refinement

**Table:**

Refinement on *F*^2^	Secondary atom site location: difference Fourier map
Least-squares matrix: full	Hydrogen site location: inferred from neighbouring sites
*R*[*F*^2^ > 2σ(*F*^2^)] = 0.057	H-atom parameters constrained
*wR*(*F*^2^) = 0.085	*w* = 1/[σ^2^(*F*_o_^2^) + (0.0112*P*)^2^ + 1.8681*P*] where *P* = (*F*_o_^2^ + 2*F*_c_^2^)/3
*S* = 1.19	(Δ/σ)_max_ < 0.001
4675 reflections	Δρ_max_ = 0.76 e Å^−^^3^
227 parameters	Δρ_min_ = −1.76 e Å^−^^3^
0 restraints	Extinction correction: *SHELXL97* (Sheldrick, 2008), Fc^*^=kFc[1+0.001xFc^2^λ^3^/sin(2θ)]^-1/4^
Primary atom site location: structure-invariant direct methods	Extinction coefficient: 0.0094 (3)

### Special details

**Table:**

Geometry. All e.s.d.'s (except the e.s.d. in the dihedral angle between two l.s. planes) are estimated using the full covariance matrix. The cell e.s.d.'s are taken into account individually in the estimation of e.s.d.'s in distances, angles and torsion angles; correlations between e.s.d.'s in cell parameters are only used when they are defined by crystal symmetry. An approximate (isotropic) treatment of cell e.s.d.'s is used for estimating e.s.d.'s involving l.s. planes.
Refinement. Refinement of *F*^2^ against ALL reflections. The weighted *R*-factor *wR* and goodness of fit *S* are based on *F*^2^, conventional *R*-factors *R* are based on *F*, with *F* set to zero for negative *F*^2^. The threshold expression of *F*^2^ > σ(*F*^2^) is used only for calculating *R*-factors(gt) *etc*. and is not relevant to the choice of reflections for refinement. *R*-factors based on *F*^2^ are statistically about twice as large as those based on *F*, and *R*- factors based on ALL data will be even larger.

### Fractional atomic coordinates and isotropic or equivalent isotropic displacement parameters (Å^2^)

**Table:**

	*x*	*y*	*z*	*U*_iso_*/*U*_eq_	
O1	0.7498 (3)	0.9688 (4)	0.04677 (14)	0.0569 (9)	
H1C	0.8214	1.0222	0.0583	0.085*	
O2	0.8222 (3)	0.8846 (4)	0.14010 (14)	0.0583 (10)	
N1	0.4007 (4)	0.6022 (5)	0.0507 (2)	0.0561 (11)	
H1B	0.3308	0.5436	0.0427	0.067*	
C1	0.4199 (5)	0.6891 (5)	0.0081 (2)	0.0527 (13)	
H1A	0.3586	0.6866	−0.0296	0.063*	
C6	0.7409 (5)	0.8855 (5)	0.0914 (2)	0.0419 (11)	
C2	0.5301 (5)	0.7820 (5)	0.01973 (19)	0.0425 (11)	
H2B	0.5451	0.8430	−0.0100	0.051*	
C5	0.4841 (6)	0.6009 (6)	0.1054 (2)	0.0586 (14)	
H5A	0.4668	0.5372	0.1338	0.070*	
C4	0.5956 (5)	0.6935 (6)	0.1197 (2)	0.0489 (12)	
H4A	0.6541	0.6947	0.1580	0.059*	
C3	0.6198 (4)	0.7850 (5)	0.07631 (18)	0.0376 (10)	
Cl5	0.21814 (12)	0.35116 (14)	0.08488 (5)	0.0501 (3)	
O1W	0.9650 (5)	0.1234 (6)	0.0754 (3)	0.195 (4)	
H1WA	1.0285	0.0715	0.0984	0.292*	
H1WB	0.9982	0.2007	0.0627	0.292*	
Sb1	0.38449 (3)	0.14611 (3)	0.173118 (11)	0.03103 (11)	
Cl1	0.51635 (14)	−0.04608 (16)	0.24298 (6)	0.0646 (4)	
Cl3	0.18317 (14)	0.09286 (16)	0.21279 (7)	0.0642 (4)	
Cl2	0.60393 (12)	0.19468 (15)	0.13735 (5)	0.0521 (3)	
Cl4	0.30831 (13)	−0.04201 (14)	0.09880 (6)	0.0554 (3)	
O3	−0.0419 (4)	0.6811 (4)	0.04218 (15)	0.0734 (12)	
H3A	−0.0944	0.6770	0.0070	0.110*	
O4	−0.1684 (4)	0.4904 (5)	0.05540 (16)	0.0797 (13)	
N2	0.1577 (4)	0.5697 (5)	0.25122 (17)	0.0513 (11)	
H2A	0.2057	0.5670	0.2877	0.062*	
C12	−0.0780 (5)	0.5765 (6)	0.0741 (2)	0.0474 (12)	
C9	0.0065 (4)	0.5778 (5)	0.13730 (19)	0.0388 (10)	
C8	−0.0139 (5)	0.4684 (6)	0.1746 (2)	0.0497 (12)	
H8A	−0.0816	0.3958	0.1610	0.060*	
C11	0.1813 (5)	0.6798 (6)	0.2169 (2)	0.0565 (14)	
H11A	0.2490	0.7516	0.2321	0.068*	
C10	0.1047 (5)	0.6868 (5)	0.1585 (2)	0.0528 (13)	
H10A	0.1190	0.7639	0.1339	0.063*	
C7	0.0648 (5)	0.4649 (6)	0.2321 (2)	0.0533 (13)	
H7A	0.0525	0.3886	0.2576	0.064*	

### Atomic displacement parameters (Å^2^)

**Table:**

	*U*^11^	*U*^22^	*U*^33^	*U*^12^	*U*^13^	*U*^23^
O1	0.051 (2)	0.072 (3)	0.046 (2)	−0.0181 (18)	0.0115 (17)	0.0049 (18)
O2	0.052 (2)	0.071 (3)	0.0415 (19)	0.0017 (18)	−0.0026 (17)	−0.0019 (17)
N1	0.060 (3)	0.047 (3)	0.062 (3)	−0.012 (2)	0.019 (2)	−0.002 (2)
C1	0.058 (3)	0.051 (3)	0.044 (3)	−0.010 (3)	0.007 (3)	−0.004 (2)
C6	0.039 (3)	0.050 (3)	0.036 (2)	0.003 (2)	0.009 (2)	−0.006 (2)
C2	0.048 (3)	0.047 (3)	0.031 (2)	−0.007 (2)	0.009 (2)	0.000 (2)
C5	0.077 (4)	0.050 (3)	0.058 (3)	0.001 (3)	0.033 (3)	0.014 (3)
C4	0.055 (3)	0.058 (3)	0.036 (3)	0.006 (3)	0.016 (2)	0.007 (2)
C3	0.040 (3)	0.044 (3)	0.032 (2)	0.004 (2)	0.015 (2)	−0.002 (2)
Cl5	0.0542 (7)	0.0498 (7)	0.0434 (6)	0.0001 (6)	0.0096 (6)	0.0118 (6)
O1W	0.074 (4)	0.146 (5)	0.316 (9)	−0.059 (4)	−0.017 (5)	0.112 (6)
Sb1	0.03618 (18)	0.02999 (17)	0.02550 (15)	0.00013 (12)	0.00678 (12)	0.00076 (12)
Cl1	0.0674 (9)	0.0654 (9)	0.0561 (8)	0.0156 (7)	0.0105 (7)	0.0324 (7)
Cl3	0.0663 (9)	0.0638 (9)	0.0750 (10)	−0.0064 (7)	0.0401 (8)	−0.0022 (7)
Cl2	0.0472 (7)	0.0707 (9)	0.0378 (6)	0.0062 (6)	0.0115 (6)	0.0107 (6)
Cl4	0.0676 (9)	0.0479 (7)	0.0506 (7)	−0.0077 (6)	0.0169 (7)	−0.0207 (6)
O3	0.094 (3)	0.070 (3)	0.041 (2)	−0.026 (2)	−0.005 (2)	0.0129 (18)
O4	0.079 (3)	0.102 (3)	0.043 (2)	−0.046 (3)	−0.006 (2)	0.007 (2)
N2	0.053 (3)	0.058 (3)	0.034 (2)	0.012 (2)	−0.0022 (19)	−0.002 (2)
C12	0.051 (3)	0.049 (3)	0.037 (3)	−0.002 (2)	0.006 (2)	−0.002 (2)
C9	0.039 (3)	0.039 (3)	0.035 (2)	0.004 (2)	0.005 (2)	−0.005 (2)
C8	0.053 (3)	0.053 (3)	0.039 (3)	−0.008 (2)	0.008 (2)	−0.003 (2)
C11	0.049 (3)	0.049 (3)	0.057 (3)	−0.004 (2)	−0.006 (3)	−0.006 (3)
C10	0.055 (3)	0.046 (3)	0.050 (3)	−0.005 (2)	0.002 (3)	0.007 (2)
C7	0.060 (3)	0.057 (3)	0.039 (3)	−0.001 (3)	0.008 (3)	0.007 (2)

### Geometric parameters (Å, °)

**Table:**

O1—C6	1.306 (5)	Sb1—Cl1	2.4661 (13)
O1—H1C	0.8500	Sb1—Cl3	2.5613 (15)
O2—C6	1.210 (5)	Sb1—Cl2	2.6748 (14)
N1—C1	1.322 (6)	O3—C12	1.306 (6)
N1—C5	1.325 (6)	O3—H3A	0.8500
N1—H1B	0.8600	O4—C12	1.180 (5)
C1—C2	1.359 (6)	N2—C7	1.307 (6)
C1—H1A	0.9300	N2—C11	1.326 (6)
C6—C3	1.484 (6)	N2—H2A	0.8600
C2—C3	1.386 (6)	C12—C9	1.493 (6)
C2—H2B	0.9300	C9—C8	1.357 (6)
C5—C4	1.366 (7)	C9—C10	1.375 (6)
C5—H5A	0.9300	C8—C7	1.365 (6)
C4—C3	1.379 (6)	C8—H8A	0.9300
C4—H4A	0.9300	C11—C10	1.375 (6)
O1W—H1WA	0.8500	C11—H11A	0.9300
O1W—H1WB	0.8499	C10—H10A	0.9300
Sb1—Cl5	2.9002 (14)	C7—H7A	0.9300
Sb1—Cl4	2.3646 (12)		
			
C6—O1—H1C	107.9	Cl4—Sb1—Cl3	90.89 (5)
C1—N1—C5	123.2 (5)	Cl1—Sb1—Cl3	88.91 (5)
C1—N1—H1B	118.4	Cl4—Sb1—Cl2	90.28 (5)
C5—N1—H1B	118.4	Cl1—Sb1—Cl2	88.01 (5)
N1—C1—C2	119.4 (5)	Cl3—Sb1—Cl2	176.73 (4)
N1—C1—H1A	120.3	C12—O3—H3A	109.1
C2—C1—H1A	120.3	C7—N2—C11	123.0 (4)
O2—C6—O1	125.3 (5)	C7—N2—H2A	118.5
O2—C6—C3	121.8 (4)	C11—N2—H2A	118.5
O1—C6—C3	112.8 (4)	O4—C12—O3	123.9 (5)
C1—C2—C3	119.4 (5)	O4—C12—C9	123.2 (5)
C1—C2—H2B	120.3	O3—C12—C9	112.9 (4)
C3—C2—H2B	120.3	C8—C9—C10	119.2 (4)
N1—C5—C4	119.9 (5)	C8—C9—C12	119.2 (4)
N1—C5—H5A	120.1	C10—C9—C12	121.6 (4)
C4—C5—H5A	120.1	C9—C8—C7	120.1 (5)
C5—C4—C3	118.7 (5)	C9—C8—H8A	119.9
C5—C4—H4A	120.6	C7—C8—H8A	119.9
C3—C4—H4A	120.6	N2—C11—C10	119.3 (5)
C4—C3—C2	119.3 (4)	N2—C11—H11A	120.3
C4—C3—C6	119.2 (4)	C10—C11—H11A	120.3
C2—C3—C6	121.5 (4)	C9—C10—C11	118.8 (5)
H1WA—O1W—H1WB	109.5	C9—C10—H10A	120.6
Cl5—Sb1—Cl1	175.24 (4)	C11—C10—H10A	120.6
Cl5—Sb1—Cl3	90.03 (4)	N2—C7—C8	119.4 (5)
Cl5—Sb1—Cl2	93.12 (4)	N2—C7—H7A	120.3
Cl5—Sb1—Cl4	84.06 (4)	C8—C7—H7A	120.3
Cl4—Sb1—Cl1	91.32 (6)		

### Hydrogen-bond geometry (Å, °)

**Table:**

*D*—H···*A*	*D*—H	H···*A*	*D*···*A*	*D*—H···*A*
O1—H1C···O1W^i^	0.85	1.67	2.520 (5)	175
N1—H1B···O4^ii^	0.86	2.45	3.031 (6)	126
O1W—H1WA···Cl3^iii^	0.85	2.71	3.378 (7)	136
O1W—H1WB···Cl5^iii^	0.85	2.54	3.241 (4)	140
O3—H3A···Cl5^ii^	0.85	2.19	3.034 (4)	175
N2—H2A···O2^iv^	0.86	2.41	2.988 (5)	125
N2—H2A···Cl2^v^	0.86	2.49	3.224 (4)	144
N1—H1B···Cl5	0.86	2.42	3.147 (4)	143

Symmetry codes: (i) *x*, *y*+1, *z*; (ii) −*x*, −*y*+1, −*z*; (iii) *x*+1, *y*, *z*; (iv) −*x*+1, *y*−1/2, −*z*+1/2; (v) −*x*+1, *y*+1/2, −*z*+1/2.
